# A New Medical Journal

**Published:** 1870-01

**Authors:** 


*A New Medical Journal.*—The first number of a new monthly Journal, under the title of *The Baltimore Medical Journal*, will be issued early in January, under the editorship of E. Lloyd Howard, M. D., and T. S. Latimer, M. D. The plan of this Journal will embrace Original Communications, Selected Articles and Translations—from home and abroad ; Reviews, and Bibliographical notices, with Editorial notes on current Medical Topics. The co-operation of some of the most talented writers of the country has been secured, and we feel certain, from the well known ability of the editors, that this new enterprise must prove a success. In form and size, we understand that the new Journal will resemble the *London Practitioner*. There is no reason why such a Baltimore Publication should not meet with favor and be largely patronized by the medical profession.

Those of our readers who do not subscribe for a Medical Journal, will find it to their advantage to become subscribers to this new Journal.

